# A Novel Multiobject Tracking Approach in the Presence of Collision and Division

**DOI:** 10.1155/2015/695054

**Published:** 2015-05-17

**Authors:** Mingli Lu, Benlian Xu, Andong Sheng, Zhengqiang Jiang, Liping Wang, Peiyi Zhu, Jian Shi

**Affiliations:** ^1^School of Automation, Nanjing University of Science & Technology, Nanjing 210094, China; ^2^School of Electrical & Automatic Engineering, Changshu Institute of Technology, Changshu 215500, China; ^3^Department of Biomedical Engineering, University of Houston, Houston, TX 77004, USA; ^4^Sansom Institute for Health Research, School of Pharmacy and Medical Sciences, University of South Australia, Adelaide, SA 5001, Australia

## Abstract

This paper aims to develop a general framework for accurately tracking and quantitatively characterizing multiple cells (objects) when collision and division between cells arise. Through introducing three types of interaction events among cells, namely, independence, collision, and division, the corresponding dynamic models are defined and an augmented interacting multiple model particle filter tracking algorithm is first proposed for spatially adjacent cells with varying size. In addition, to reduce the ambiguity of correspondence between frames, both the estimated cell dynamic parameters and cell size are further utilized to identify cells of interest. The experiments have been conducted on two real cell image sequences characterized with cells collision, division, or number variation, and the resulting dynamic parameters such as instant velocity, turn rate were obtained and analyzed.

## 1. Introduction

Being the fundamental unit of life, cell is a key element in many biological processes. Researchers have realized the importance of studying cells motility, deformation or population dynamics, and cell to cell interactions in modern biology. Understanding the dynamical behaviors of cell of interest in living cells is essential to the fundamental studies for discovering effective medical therapy of diseases like cancer, AIDS, or any inflammatory diseases [1]. Manual analysis of these images is a tedious process involving many hours of human inspection. Sometimes, it becomes impossible for the human observer to accurately follow many different events over a long sequence, especially when it requires tracking a large number of cells during long period of time in order to obtain robust results [2]. This makes automatic cell motion analysis essential. However, these tasks face several challenges including the generally poor image quality (low-SIR), the varying cell populations due to cells entering or leaving the field of view, and the possibility of irregular interaction among cells.

Over the past decade, a number of cell tracking algorithms have been proposed (see [3] for a review). These algorithms concentrate on a variety of cell types and are based on different tracking methods. These cell tracking approaches in the literature can be broadly classified into three categories, namely, tracking based on detection and segmentation [4–6], tracking based on evolving model [7–9], and tracking based on probabilistic approach [10–13].

The first type of approaches is to run a cell detector based on texture, intensity, or gradient in every frame and then associate the detected cells between frames [14]. It is noted that the tracking performance mainly depends on the quality of detection and segmentation and sophisticated matching strategies [15–17]. The second approach is to initialize the features of cells such as shape, position, and boundary and then track them using an appropriate tracking technique. Active contour [18, 19], level set [16, 20, 21], and mean-shift are the examples of this type of approach. As the classic mean-shift or active contour technique is designed for tracking single object only, cell clusters may cause matching errors and inaccurate boundaries when cells move fast. Although the level set method enables the topological changing for cell division and details permit the fusion of overlapping cells, its computation time is expensive [5]. For the third type of the approaches, the probabilistic framework has also been increasingly used (see [22–25] for reviews in this area). Probabilistic tracking methods, that is, Bayesian filtering, usually construct a motion evolution model described by a Markov process, and then track multiple cells using the filtering techniques such as GM-PHD [26] and particle filter [10]. The first two approaches render more robust performance than the third approach under low resolution or SNR scenarios, but more modifications and assumptions are needed in general.

Inspired by work in [27], where the cell collision is only considered, we propose a more general framework for multicell tracking especially when cell collision and division occur. We first define three typical events to characterize interaction modes among cells, that is, independence, collision, and division. Afterwards, the evolving model relevant to each event is described and an augmented interacting multiple models particle filter tracking algorithm is proposed for spatially adjacent cells with varying size. Finally, to reduce the ambiguity of correspondence and establish trajectories of interested cells, both cell topological feature and cell motion feature are used to manage data association problem.

This rest of the paper is organized as follows.  Section 2 presents our proposed general framework for multicell tracking including cell collision and division. In Section 3, the experiment results on various real cell image sequences are presented to demonstrate the effectiveness of our algorithm. Finally, conclusions are summarized in Section 4.

## 2. Methods

This section describes our proposed method in detail. In Section 2.1, a hybrid cell detection algorithm for image sequences is presented. In Section 2.2, we first define and analyze three typical interacting events between cells and the corresponding dynamic model of each cell is formulated. In Section 2.3, the augmented interaction multiple models particle filter (AIMMPF) tracking algorithm is proposed to deal with all interacting cases happening among cells. Finally, the cell identity management for cell collision and division is presented in Section 2.4.

### 2.1. A Hybrid Cell Detection Algorithm for Image Sequences

**Figure 1:**
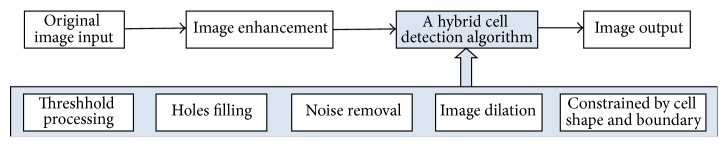
A block diagram of the proposed cell detection method.

The cell detection is a challenging job due to a high noise level in time-lapse microscopy images and wide ranging in intensity and shape. Image enhancement removes blurring and noise, increases contrast, and so forth. After the process of image enhancement, “a hybrid cell detection algorithm” is used to segment overlapping or adhesion cells. This combination method consists of threshold processing, holes filling, noise removal, image dilation, and shape and boundary constraint. The overview of the proposed detection method is given in Figure 1.

#### 2.1.1. A Hybrid Cell Detection Algorithm

During the threshold process, individual pixels in an image are marked as “object” pixels if their values are greater than some threshold value (assuming an object to be brighter than the background) and as “background” pixels otherwise. Typically, an object pixel is given a value of “1” while a background pixel is given a value of “0.” Finally, a binary image is created. The key parameter in the threshold process is the choice of the threshold value. Since *K*-means clustering algorithm does not require much specific knowledge of the image and is robust to image noise, it converts a grayscale image to a binary image. The description of threshold process can be briefly presented as follows.

**Step 1 .**

An initial threshold (TH) is chosen, and this can be done randomly or according to any other methods desired.

**Step 2 .**

Create two sets

(1) G1fm,n:fm,n>TH Object pixels,G2=fm,n:fm,n≤TH Background pixels.

Note, *f*(*m*, *n*) is the value of the pixel located in the *m*th column, *n*th row.

**Step 3 .**

Compute the average intensity of each set

(2) m1Average intensity value of  G1,m2=Average intensity value of  G2.

**Step 4 .**

Create the new threshold

(3) $$\begin{array}{c} {\text{TH}}^{\prime }=\frac{\left(m_{1}+m_{2}\right)}{2}. \end{array}$$

**Step 5 .**

Go back to Step 2 until convergence is reached.

**Figure 2:**
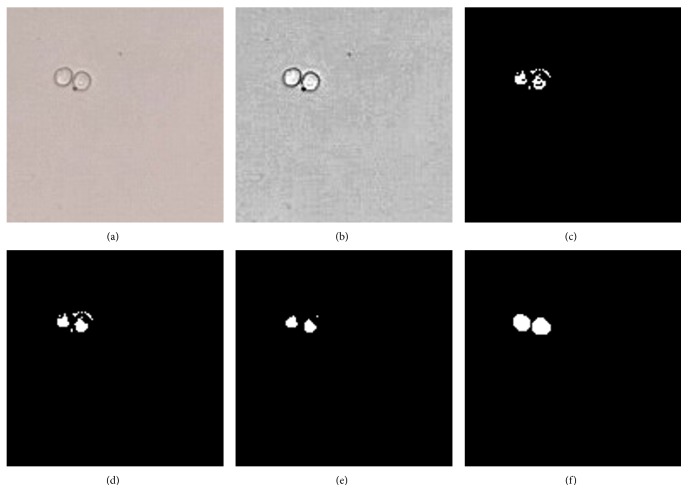
Illustration of the adhesion cell detection by proposed method. (a) Original image, (b) the contrast enhancement results on the whole image, (c) threshold process image, (d) hole-filled image, (e) noise removal image using median filter, and (f) result of our algorithm.

As shown in Figure 2(c), most cell edges are broken and have holes, to handle this problem the broken mending and holes filled algorithm is required. The concept of mending cell edges is that the missed pixel can be retrieved by the surrounding pixels. Given a binary image, the value of a single pixel only is taken as 1 or 0. If the sum of all these 3 pixels equals 2 in any direction (horizontal, vertical, or diagonal), then the centre pixel will be corrected from 0 to 1. A hole is a set of background pixels (0's or blacks in binary image). Holes filled algorithm is to connect background pixels (0's) need to be changed to foreground pixels (1's) and stopping when it reaches cell boundaries. This can be achieved using morphological reconstruction which can be thought conceptually as repeated dilations of an image, called the marker image, until the contour of the marker image fits under a second image, called the mask image. We set a pixel of marker image to 1 if its corresponding background pixel in mask image cannot be reached by filling background pixels from borders of mask image. And all other pixels are set to be 0. Processing repeats until stability; that is, the image no longer changes. By using this method, only holes in the foreground are filled. Then we add marker image to the mask image to get the holes filled image. The zoomed in hole-filled image is obtained in the testing result as shown in Figure 2(d).

By a careful observation of image, the unexpected high frequency noise significantly affects the quality of image. A median filter is employed to perform noise removal. Median filter runs through the image pixel by pixel and replaces each pixel with the median of neighbor pixels, which is also known as a smoothing technique. To minimize the distortion of edges and remove noise effectively, the value of threshold should be considered carefully. If the threshold of median filter is set a bit low from the value, all the noise can be removed.  Figure 2(e) shows after high frequency noise removal. It is obvious that the cell contour profile is smaller than normal. So, dilation is proposed to enlarge the cells.

After applying dilation filter, the boundaries of regions of foreground pixels which are typically bright pixels in the image are gradually enlarged. The areas of foreground pixels grow in size. Shape and boundary constraint is finally proposed and used to discriminate cells. If a component is either smaller than minimum, or bigger than maximum of cell size range, it will be removed as a noncell component; otherwise it will be kept as cell component. After setting up a bounding box to calculate the cells area, we continue to calculate the “width/height ratio,” and remove the component whose ratio is either bigger than 5.0 or less than 0.5; both are determined empirically.

Figure 2(f) shows the result of our algorithm.

### 2.2. Events and Dynamic Model

#### 2.2.1. Events

**Figure 3:**
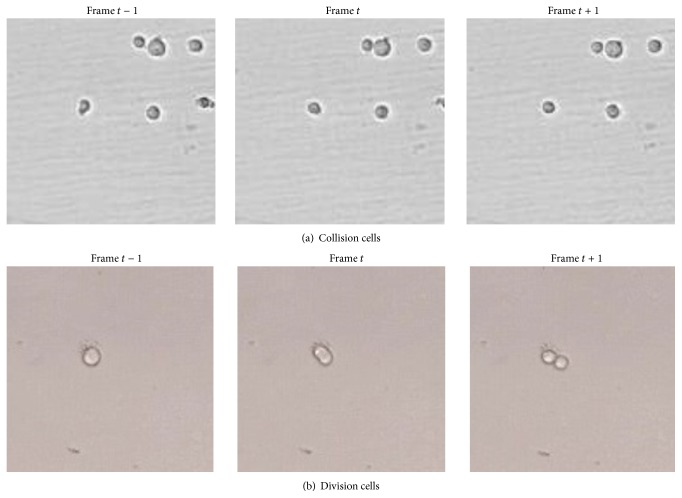
An illustration of collision and division cells.

In the field of cell tracking, each cell exhibits various behaviors, such as random moving fluctuation, collision, division, and shape deformation in different frames, as illustrated in Figure 3, and the last three challenging cases obviously differ from the general people tracking in computer vision, which further complicate the multicell accurate and robust tracking. However, from the perspective of interacting way among cells, multicell tracking can be explained by three interacting events among cells; namely, one cell evolves independently without any interaction with other cells; one cell collides with other cells for one or more frames; and one cell or merged cell divides into two or more individual cells in the next frame. Accordingly, we define three events, that is, independence event, collision event, and division event.

*Independence Event*. In terms of the independence event, a given cell is assumed to move in a random way but does not undergo collision or division in any frame, which is the simple and common case in people and/or cell tracking. The state space tracking techniques, such as the Karman filter and the particle filter, are the commonly used methods.

**Figure 4:**
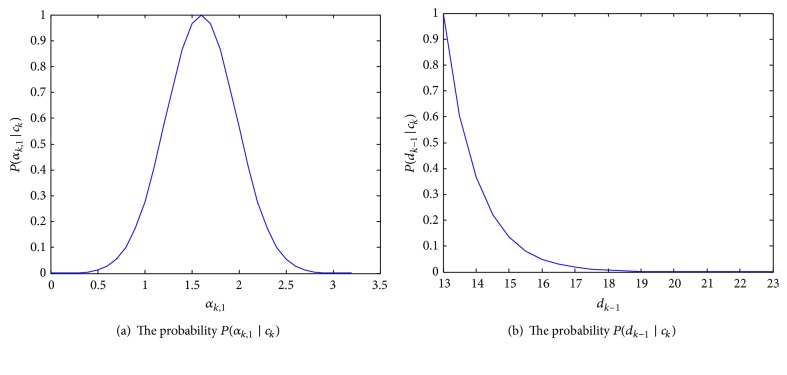
The distribution of two elements of cell collision event.

*Collision Event*. Without loss of generality, suppose that a collision event *c*_*k*_ between two cells occurs in frame *k*, and *P*(*c*_*k*_) denotes the probability of the collision event. Consider morphological features area and distance. Given two independent variables *α*_*k*,1_ and *d*_*k*−1_, *P*(*c*_*k*_) can be calculated as follows:

(4) $$\begin{array}{c} P\left(\;c_{k}\;\right)=f_{1}*P\left(\;\alpha_{k,1}\;|\;c_{k}\;\right)P\left(\;d_{k-1}\;|\;c_{k}\;\right), \end{array}$$

where *f*_1_ is an adjusted coefficient, *α*_*k*,1_ = *s*_*k*_/*s*_*k*−1_ denotes the ratio of a given cell area in the current frame *k* to the one in the previous frame *k* − 1, *s*_*k*_ is the detected area of a given cell in frame *k*, and *d*_*k*−1_ is the distance of two cells in frame *k* − 1. *P*(*α*_*k*,1_∣*c*_*k*_) defines the probability of area ratio given the event of collision occurring. For simplicity, we assume that *P*(*α*_*k*,1_∣*c*_*k*_) follows the Gaussian distribution with *P*(*α*_*k*,1_∣*c*_*k*_) ∝ (*α*_*k*,1_ : *μ*_1_, *σ*_1_^2^), as shown in Figure 4(a), where the mean *μ*_1_ and *σ*_1_^2^ are determined empirically based on cell collision event. In terms of the probability *P*(*d*_*k*−1_∣*c*_*k*_), due to the fact that the distance between two cells will decrease at collision or increase at splitting, we have *P*(*d*_*k*−1_∣*c*_*k*_) = *e* ^−*σ*|*d*_*k*−1_|^, as shown in Figure 4(b), where *σ* is a constant and obtained according to the average size of studied cell population.

**Figure 5:**
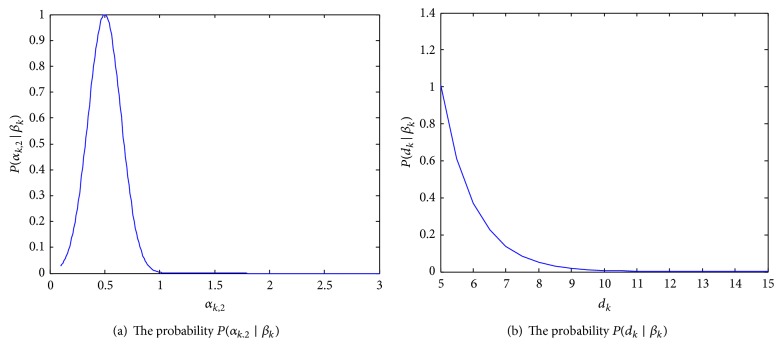
The distribution of two elements of cell fission event.

*Division Event*. Assume that a cell divides into two cells or colliding cells split away from one another in frame *k*, and the division event is denoted by *β*_*k*_. Given two independent variables *α*_*k*,2_ and *d*_*k*_, the possibility *P*(*β*_*k*_) of division event is calculated as follows:

(5) $$\begin{array}{c} P\left(\;\beta_{k}\;\right)=f_{2}*P\left(\;\alpha_{k,2}\;|\;\beta_{k}\;\right)P\left(\;d_{k}\;|\;\beta_{k}\;\right), \end{array}$$

where *f*_2_ is an adjusted coefficient, *α*_*k*,2_ follows the same definitions as in (4), and *d*_*k*_ is the distance of two cells in the presence of division event in frame *k*. *P*(*α*_*k*,2_∣*β*_*k*_) defines the probability of area ratio given the division event. Since the expectation of variable *α*_*k*,2_ is equal to 0.5, we model this as *P*(*α*_*k*,2_∣*β*_*k*_) = *e* ^−*C*(*α*_*k*,2_−0.5)^2^^, as shown in Figure 5(a), where *C* is a constant. According to the evolvement of spatial distance between cells in the division event, we define *P*(*d*_*k*_∣*β*_*k*_) = *e* ^−*γ*|*d*_*k*_|^, as shown in Figure 5(b), where *γ* is a constant and obtained according to the average size of studied cell population.

#### 2.2.2. Dynamic Model

The IMMPF framework [28, 29] facilitates multiple models [30] to be combined, with each model corresponding to a standard particle filter [31]. The IMMPF is expected to be effective in dealing with nonlinear cell parameter estimate as multiple motion models can be accommodated and switching between motion models can be designed using a predefined Markov switching process.

Assume that there are total *D* cells with the cell set denoted as Γ_*D*_ : = {1,2,…, *D*} and the behaviors of each cell can be modeled as one of the *n* hypothesized modes. The mode set is denoted as *M*_*n*_ : = {1,2,…, *n*}. For cell *r*  (*r* ∈ Γ_*D*_), let the probability that the *i*th mode is relevant in frame *k* be denoted by *M*_*k*_^*i*^(*r*). For the *j*th mode, the state dynamics and measurements of cell *r*  (*r* ∈ Γ_*D*_) are modeled as

(6) XkrFk,jrXk−1r+Gk,jVk,jr,

(7) Zkr=Hk,jrXkr+wk,jr,

where $X_{k}(r)={\left[x_{k}\left(r\right),y_{k}\left(r\right),{\dot{x}}_{k}\left(r\right),{\dot{y}}_{k}\left(r\right)\right]}^{T}$ is the state vector of each cell for each mode is represented by the position (*x*_*k*_(*r*), *y*_*k*_(*r*)) and the velocity $\left({\dot{x}}_{k}^{}(r),{\dot{y}}_{k}^{}(r)\right)$. *F*_*k*,*j*_(*r*) and *G*_*k*,*j*_(*r*) are the system matrices when mode *j* is in effect in frame *k* for cell *r*. Both *v*_*k*,*j*_(*r*) and *w*_*k*,*j*_(*r*) are covariance matrices *Q*_*k*,*j*_ and *R*_*k*,*j*_ (same for all cells), respectively. The switching from model *M*_*k*−1_^*i*^(*r*) to model *M*_*k*_^*j*^(*r*) is governed by a finite-state stationary Markov chain (same for all cells) with known transition probabilities *P*_*i*,*j*_ = *P*(*M*_*k*_^*j*^(*r*)∣*M*_*k*−1_^*i*^(*r*)).

### 2.3. Cell State Evolvement in Augmented IMMPF

In this section, to characterize cell dynamics and quantitative study multicell behaviors, we propose an augmented interacting multiple models particle filter (AIMMPF) tracking algorithm to accurately estimate the state vectors of multiple cells.

#### 2.3.1. Cell State Evolving Modes

As observed in a series of cell image sequences, some cells exhibit unpredictable behaviors when cell collision and division occur, such as sudden change in motion speed, direction, and size of cell area. To deal with these uncertainties, we augment the state vectors in (6) by the unknown cell turn rate *ω*_*k*_(*r*) and cell area *s*_*k*_(*r*), resulting in $X_{k}(r)={\left[x_{k}(r),y_{k}(r),{\dot{x}}_{k}(r),{\dot{y}}_{k}(r),\omega_{k}(r),s_{k}(r)\right]}^{T}$.

We also observe that a successful implementation of our proposed AIMMPF relies on two aspects, namely, the determination of turn rate *ω*_*k*_(*r*) and the way of modeling cell interaction modes.

In terms of the turn rate variable *ω*_*k*_(*r*), assume that system dynamic follows

(8) $$\begin{array}{c} \omega_{k,m}^{}\left(\;r\;\right)=\omega_{k-1,m}^{}\left(\;r\;\right)+\delta_{k-1,m}^{}\left(\;r\;\right), \end{array}$$

where *ω*_*k*,*m*_(*r*)  (*m* = 1,2) is the turn rate in radians/second. Modes based on different *ω*_*k*,*m*_(*r*) can match different motions of a cell. A positive value of *ω*_*k*,*m*_(*r*) can be assumed for right turn and a negative value for left turn. *δ*_*k*−1,*m*_ is assumed to be a Gaussian distributed noise with covariance Δ_*k*−1,*m*_. In our algorithm, variable *ω*_*k*,*m*_(*r*) is determined at the end of previous *k* − 1 scan upon the base of *ω*_*k*−1,*m*_(*r*).

Another key issue in our proposed algorithm is how to determine the evolvement of cell state at each mode. Without loss of generality, we propose three interaction modes for cell tracking, namely, augmented variable nearly constant velocity mode for cell noninteracting (ACV), an augmented variable coordinate turn mode for cell collision (ACT1), and an augmented variable coordinate turn mode for cell division (ACT2).

**Mode 1 (ACV).**

If a given cell does not undergo collision or division, in other words, it moves smoothly with minimum appearance change, the following state transition model is adopted:

(9) $$\begin{array}{c} F_{k,1}^{}\left(\;r\;\right)=\left[\begin{array}{cccccc} 1 & 0 & T & 0 & 0 & 0\\ 0 & 1 & 0 & T & 0 & 0\\ 0 & 0 & 1 & 0 & 0 & 0\\ 0 & 0 & 0 & 1 & 0 & 0\\ 0 & 0 & 0 & 0 & 1 & 0\\ 0 & 0 & 0 & 0 & 0 & 1 \end{array}\right], \end{array}$$

where *T* denotes the sampling interval, *v*_*k*,1_ is assumed to be zero mean Gaussian white noise with 6 × 6 covariance $Q_{k,1}=\mathrm{diag}(\sigma_{x,k,1}^{2},\sigma_{y,k,1}^{2},\sigma_{\dot{x},k,1}^{2},\sigma_{\dot{y},k,1}^{2},\sigma_{\omega ,k,1}^{2},\sigma_{s,k,1}^{2})$, and the noise transition matrix is given as

(10) $$\begin{array}{c} G_{k,1}={\left[\;\begin{array}{cccccc} \frac{1}{2}T^{2} & 0 & T & 0 & 0 & 0\\ 0 & \frac{1}{2}T^{2} & 0 & T & 0 & 0 \end{array}\;\right]}^{T}. \end{array}$$

**Mode 2 (ACT1).**

If one cell collides with the other in a given frame, its motion speed, direction, and area are assumed to vary accordingly. The current cell turn rate is fluctuant and unknown to us, but we can use (8) to approximately estimate the turn rate. Meanwhile, cell area will increase as well, but it does not exceed the sum of two colliding cells. Thus, the following state evolvement is formulated as

(11) Fk,2r=10sin⁡ωk,1rTωk,1cos⁡⁡ωk,1rTωk,1f11,k0011−cos⁡⁡ωk,1rTωk,1sin⁡ωk,1rTωk,1f21,k000cos⁡⁡ωk,1rT−sin⁡ωk,1rTf31,k000sin⁡ωk,1rTcos⁡⁡ωk,1rTf41,k000001000000λk,

where the nonlinear system Jacobi term *M*_1,*k*_ = [*f*_11,*k*_, *f*_21,*k*_, *f*_31,*k*_, *f*_41,*k*_]^*T*^ [32] is defined in the same way as

(12) f11,k=x˙kωk,1rTcos⁡⁡ωk,1rT−sin⁡ωk,1rTωk,12r−y˙kωk,1rTsin⁡ωk,1T−1+cos⁡⁡ωk,1rTωk,12r,f21,k=x˙kωk,1rTsin⁡ωk,1T+cos⁡⁡ωk,1rT−1ωk,12r−y˙kωk,1Tcos⁡⁡ωk,1rT−sin⁡ωk,1rTωk,12r,f31,k=−Tx˙ksin⁡ωk,1rT+y˙kcos⁡⁡ωk,1rT,f41,k=Tx˙kcos⁡⁡ωk,1rT−y˙ksin⁡ωk,1rT.

In addition, we further assume that $\omega_{k,1}(r)={\hat{\omega }}_{k-1,1}(r)$ and *G*_*k*,2_(*r*) = *G*_*k*,1_(*r*) in our experiment, and *v*_*k*,2_ is assumed to be zero mean Gaussian white noise with 6 × 6 covariance $Q_{k,2}=\mathrm{diag}(\sigma_{x,k,2}^{2},\sigma_{y,k,2}^{2},\sigma_{\dot{x},k,2}^{2},\sigma_{\dot{y},k,2}^{2},\sigma_{\omega ,k,2}^{2},\sigma_{s,k,2}^{2})$. The control variable *λ*_*k*_ varies in time and mainly considers the effect of merging event on the resulting size of cell, which leads to the value of *λ*_*k*_ distributed in the range [1, 2] and the centroid distance between the two cells is approximately equal to the averaged diameter of cell population. In this way, we define that the control variable as *λ*_*k*_ = 1 + *P*(*c*_*k*_) and the evolving curve for colliding cells are investigated in the following experiments.

**Mode 3 (ACT2).**

In frame *k*, if a cell is divided into two cells or the colliding cells split away from one another, each cell moves independently and appears as an individual one. Accordingly, both the velocity and area of interested cells might change from the previous frame to the current frame. Following the same rule as ACT1, we define the state transition mode as

(13) Fk,3r=10sin⁡ωk,2rTωk,2cos⁡⁡ωk,2rTωk,2f12,k0011−cos⁡⁡ωk,2rTωk,2sin⁡ωk,2rTωk,2f22,k000cos⁡⁡ωk,2rT−sin⁡ωk,2rTf32,k000sin⁡ωk,2rTcos⁡⁡ωk,2rTf42,k000001000000κk.

In terms of the nonlinear system Jacobi term *M*_2,*k*_ = [*f*_12,*k*_, *f*_22,*k*_, *f*_32,*k*_, *f*_42,*k*_]^*T*^, we follow the same formula as (12) as long as the turn rate *ω*_*k*,1_(*r*) in each corresponding component is replaced by *ω*_*k*,2_(*r*). Similarly, we have $\omega_{k,2}(r)={\hat{\omega }}_{k-1,2}(r)$ and *G*_*k*,3_(*r*) = *G*_*k*,1_(*r*). *v*_*k*,3_ is also assumed to be zero mean Gaussian white noise with 6 × 6 covariance $Q_{k,3}=\mathrm{diag}(\sigma_{x,k,3}^{2},\sigma_{y,k,3}^{2},\sigma_{\dot{x},k,3}^{2},\sigma_{\dot{y},k,3}^{2},\sigma_{\omega ,k,3}^{2},\sigma_{s,k,3}^{2})$. Note that the time-varying control variable *κ*_*k*_ takes into account the effect of division or splitting event on the resulting cell area, which leads to the value of *κ*_*k*_ in the range [0,1]. In this way, we directly define that the control variable as *κ*_*k*_ = *P*(*β*_*k*_) and the evolving curve for division cells are investigated in the following experiments as well.

#### 2.3.2. The Framework of Augmented IMMPF

**Figure 6:**
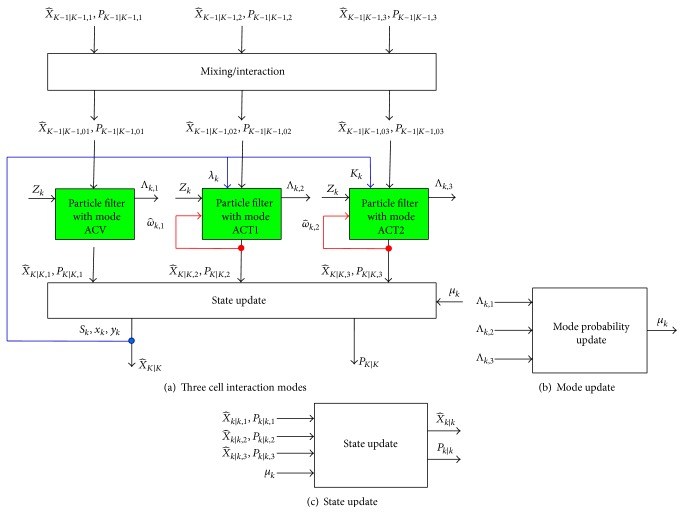
Tracking block of each cell (for simplicity, the superscript of target is omitted).

To view our proposed cell interaction based AIMMPF tracking algorithm in a straight way, Figure 6 illustrates our tracking frame work with three cell interacting modes. The red line feedback mechanism is introduced via instant turn rate estimate $\hat{\omega }$ and blue line feedback mechanism is introduced to obtain the control variables *λ*_*k*_ and *κ*_*k*_, which are drastically different from traditional IMMPF.

### 2.4. Cell Correspondence and Label Management

To track and discriminate simultaneously multiple cells, the correspondence and label management block is required for our proposed cell interacting based AIMMPF filter. The correspondence aims to introduce the measures to be associated, whereas identity management focuses on the strategy to discriminate and label each cell of interest. Therefore, we need to (1) define a dissimilarity measure between two cells in two consecutive frames and (2) design an appropriate identity management strategy.

#### 2.4.1. Dissimilarity Measure

If a cell moves in a smooth way, which means that the dynamics of the cell can be known a priori, the position of the cell in the next frame is predicted and further associated preferably with the available closest measurement (i.e., nearest neighbor method). In this way, the obtained distance difference is the smallest one, and such measure is denoted by

(14) $$\begin{array}{c} d_{k}^{\text{distance}}=\left|\;x_{k\;|\;k-1}^{}\left(\;i\;\right)-Z_{k}\;\right|, \end{array}$$

where *Z*_*k*_ is the detection measurement vector and **x**_*k*∣*k*−1_(*i*) is state prediction of cell *i*.

This function is independent of the direction of motion and allows nonsmooth trajectories. It can be seen that the above method is mainly dependent on the assumed dynamics of cell, and it often leads to tracking failures in a dense clutter environment or in the case of cell collision. Thus, we further introduce another measure, namely, area difference *d*_*k*_^area^. This parameter measures the equivalent degree of size between the predicted area of the cell *i* in frame *k* − 1 and the interested area corresponding to measurement *j* in frame *k*, which can be denoted by

(15) $$\begin{array}{c} d_{k}^{\text{area}}=\left|\;{\hat{s}}_{k\;|\;k-1}^{}\left(\;i\;\right)-s_{k}^{}\left(\;j\;\right)\;\right|, \end{array}$$

where *s*_*k*_(*j*) is interested area corresponding to measurement *j* in frame *k* and ${\hat{s}}_{k|k-1}^{}(i)$ is predicted area of the cell *i* in frame *k* − 1.

#### 2.4.2. Scheme

The direct objective in multicell tracking is to discriminate and record the dynamic parameters and feature parameters of each cell in each frame for further biological process analysis. In our study, the cell division and collision are investigated. Thus the uncertainties in correspondence increase, which further lead to the difficulty in cell label management. Since our algorithm belongs to the type of probabilistic approach with reliable detection results, both the spatiotemporal and feature information could be utilized as the inputs to correspondence and label management block. In the case of cells collision and division, cells are easily merged in one frame and separated in another frame. As a result, one of them would be detected as a newly born cell for cell division and would be assigned a new label as a new track. In addition, in the case of cell collision, two cells would be merged as one cell, due to only one detection generated around the predicted cell state. To solve this problem, three cases are investigated, and related strategy is adopted as below.

**Case 1 .**

**Figure 7:**
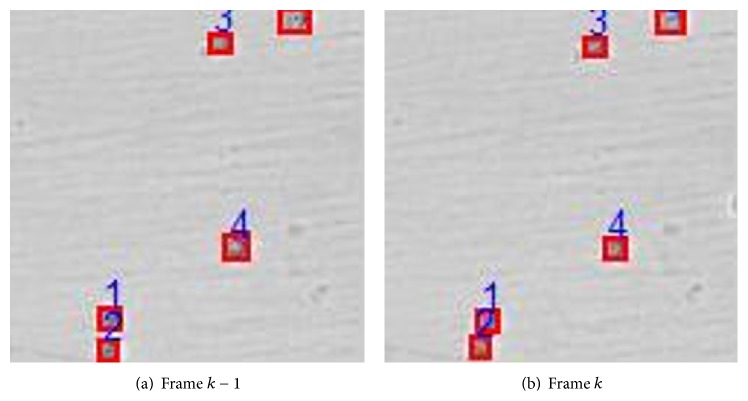
No cell collision or division.

If there is more than one measurement in the associated gate (*d*_*k*_^distance^ < *δ*_1_, *δ*_1_ is threshold), the cell feature information, such as the area, is used to be associated with the predicted state in order to reduce correspondence uncertainty. As shown in Figure 7, we assume that all cells do not undergo collision or division in frame *k*, and the area difference *d*_*k*_^area^ between the areas of the cell *i* in *k* − 1 frame and measurement *j* in frame *k* is calculated according to (15). If *d*_*k*_^area^ < *δ*_2_ (threshold), the measurement *j* in the current frame is associated with cell *i* in the previous frame.

**Case 2 .**

**Figure 8:**
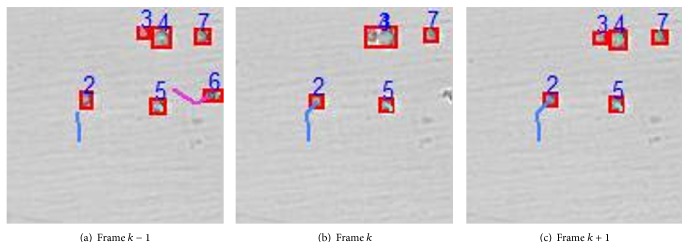
Cell collision.

Suppose that cells *i*_1_ and *i*_2_ in frame *k* − 1 collide and/or merged into one cell *i*_1,2_ in frame *k*; then the merged cell *i*_1,2_ splits into cells *j*_1_ and *j*_2_, respectively, in frame *k* + 1. In general, the two splitting cells are very close to each other, and one of cells would be probably mistaken as a new one and a new label is assigned accordingly. To solve this bias, the information of both cell area ratio, that is, *α*_*k*,1_ and *α*_*k*,2_, and cell area difference, that is, *d*_*k*_^area^, is considered. For instance, if *α*_*k*,1_(*i*_1,2_) ≈ 2, cell *i*_1,2_ in frame *k* is associated with both cell *i*_1_ and cell *i*_2_ in frame *k* − 1. In frame *k* + 1, if *α*_*k*,2_(*j*_1_) ≈ 0.5 or *α*_*k*,2_(*j*_2_) ≈ 0.5, the area difference *d*_*k*+1_^area^ is then calculated. Moreover, if *d*_*k*+1_^area^(*j*_•_, *i*_•_) < *δ*_2_, then cell *j*_•_ is associated with cell *i*_•_.  Figure 8 illustrates a successful example of our proposed cell correspondence and label approach. From Figures 8(b) and 8(c), we can see that cells 3 and 4 collide in frame *k* and then separate in frame *k* + 1.

**Case 3 .**

**Figure 9:**
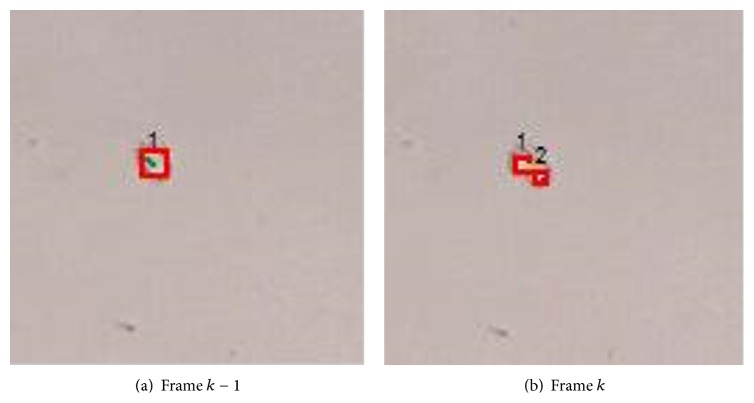
Cell fission.

Without loss of generality, we give an example of cell division as illustrated in Figure 9. Assume that cell *j* is divided into two cells *j*_1_ and *j*_2_ in frame *k*, and the detected area of cell *j*_1_ or *j*_2_ is about half of cell *j* in frame *k* − 1. If *α*_*k*,2_(*j*_1_) ≈ 0.5 or *α*_*k*,2_(*j*_2_) ≈ 0.5, both cells *j*_1_ and *j*_2_ in frame *k* are associated with cell *j* in frame *k* − 1.

**Figure 10:**
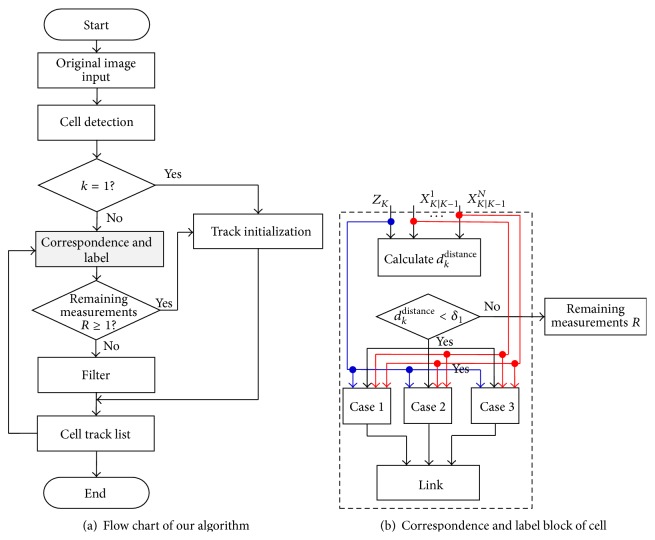
The main framework of our proposed algorithm.

The procedure of correspondence and label management scheme, which is appropriately embedded in our algorithm, is illustrated in Figure 10(a), and the detailed implementation flowchart is shown in Figure 10(b).

## 3. Results and Discussion

In this section, several experiments were conducted on two real image sequences to verify the effectiveness of our proposed method for cell tracking. These experiment data include various challenging scenarios, such as variation in cell dynamics and population, cell collision, and division in different frames. Our purpose is to estimate the position, velocity, turn rate of each cell from available cell detections. All experiments were implemented in MATLAB on a 1.7 GHz processor computer with 4G random access memory.

In our experiment, all cells are identified by rectangular blobs, and three modes in our proposed cell interacting AIMMPF are adopted, namely, ACV, ACT1, and ACT2.

**Scenario 1 .**

This case includes cell collision and variation in population, and the Markov transition matrix between three modes is assumed constant and set empirically as $M=\left[\begin{array}{ccc} 0.8 & 0.1 & 0.1\\ 0.1 & 0.8 & 0.1\\ 0.1 & 0.1 & 0.8 \end{array}\right]$; *Q*_*k*,1_ = diag(30,30,0.1,0.1,0.01,0.01); *Q*_*k*,2_ = diag(40,40,0.1,0.1,0.01,0.01); *Q*_*k*,3_ = diag(40,40,0.1,0.1,0.01,0.01); *R* = diag(1,1, 1); *δ*_1_ = 25; *δ*_2_ = 8; *T* = 1; *N* = 500; $H_{k,•}={\left[\begin{array}{ccc} 1\;0\;0\;0\;0\;0 & 0\;1\;0\;0\;0\;0 & 0\;0\;0\;0\;0\;1 \end{array}\right]}^{T}$. The initial state of cell *i* is represented as $X_{0}^{}(i)={\left[x_{0}^{}(i),y_{0}^{}(i),{\dot{x}}_{0}^{}(i),{\dot{y}}_{0}^{}(i),\omega_{0}^{}(i),s_{0}^{}(i)\right]}^{T}$, where *x*_0_(*i*), *y*_0_(*i*), and *s*_0_(*i*) were determined by the initially detected cells. The initial velocities in *x* and *y* directions were set zero, and we also set *ω*_0_(*i*) = 0.8 rad/s. In the IMMPF algorithm, all parameters were set the same values as those in our proposed approach except *ω* = 0.8 rad/s, *λ*_*k*_ = 1.6, and *κ*_*k*_ = 0.5.

**Figure 11:**
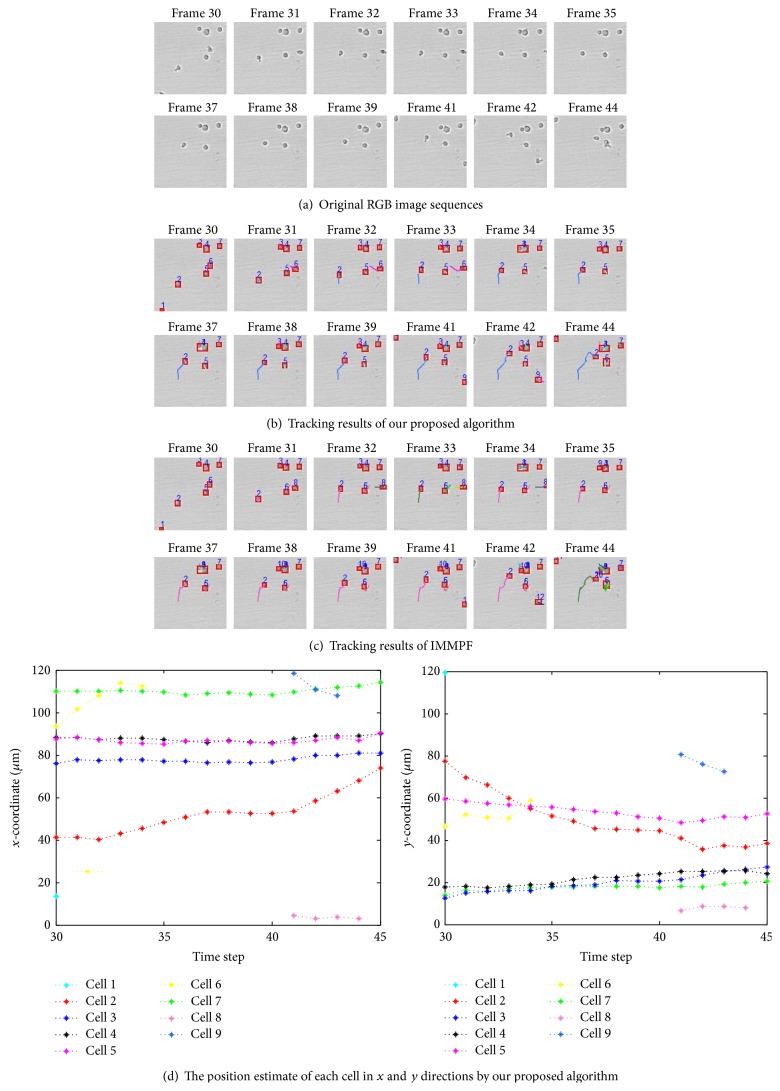
Multicell tracking with colliding and varying number of cells in different frames.

Figure 11(b) shows the successful tracking results of our proposed method on the original RGB cell image sequences (see Figure 11(a)). According to the tracking results, our algorithm could handle the following challenging cases: cell 3 collides with cell 4 in frame 34 and then splits away in frame 35; cell 3 collides again with cell 4 in frame 37 and splits in frame 38; cell 1 leaves the field of view in frames 30; cell 6 moves right, partially leaves the field of view in frames 33, and fully leaves the field of view in frame 34; and new cells 8 and 9 enter the field of view in frame 41. In terms of cell collision, splitting, and varying number, the performance is degraded when the general IMMPF is used, as shown in Figure 11(c). Cell 3 moves closely to cell 4 from frame 30 and two cells are merged together as a cell in frame 34. A new cell 9 is wrongly initiated when the detected two cells split away in frame 35, and the original cells 3 and 4 are merged together as a cell. Afterwards, the similar results occur in frames 37 and 38.  Figure 11(d) further gives the position estimate of each cell in each frame in *x* and *y* directions, respectively, using our algorithm.

**Figure 12:**
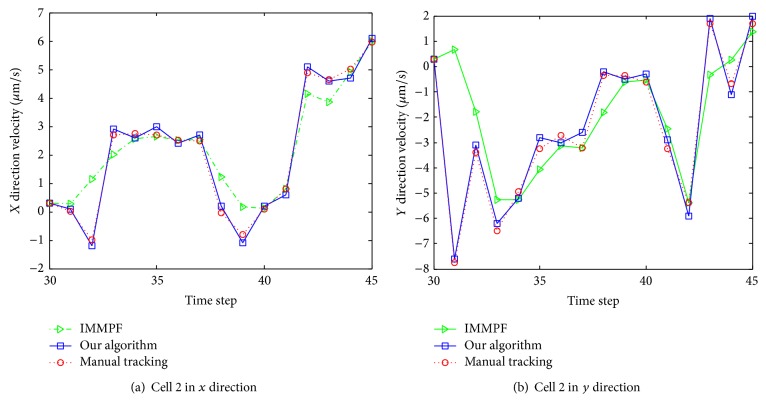
Instant velocity estimate per frame using various methods.

Due to lack of velocity ground-truth data, we evaluated the performance of our algorithm by comparison with manual tracking results. Instant velocity estimate of cell 1 per frame among three approaches is shown in Figure 12. The green dash dot line represents the instant velocity of the IMMPF, the red dot line plots the instant velocity of the manual tracking, and the blue solid line shows instant velocity curve of our proposed algorithm. From this figure we can see that our tracking method outperforms one that uses the IMMPF. The difference between our proposed algorithm and manual tracking is very small.

**Figure 13:**
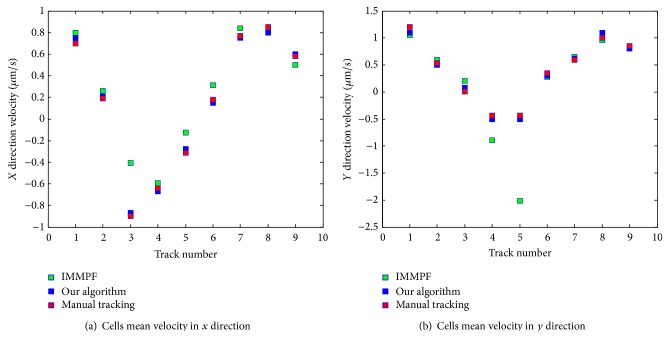
Mean velocity estimate of all cells in image using various methods.

Mean velocity (the track length is 30 frames) of all cells for our proposed algorithm versus IMMPF and manual tracking is shown in Figure 13. The green square shows all cells mean velocity of IMMPF, the red square presents all cells mean velocity of manual tracking, and the blue square describes all cells mean velocity of our proposed algorithm. The estimate error using the IMMPF is larger than our proposed algorithm. It also shows that the mean velocity precision of our proposed algorithm is higher than that of IMMPF.

**Figure 14:**
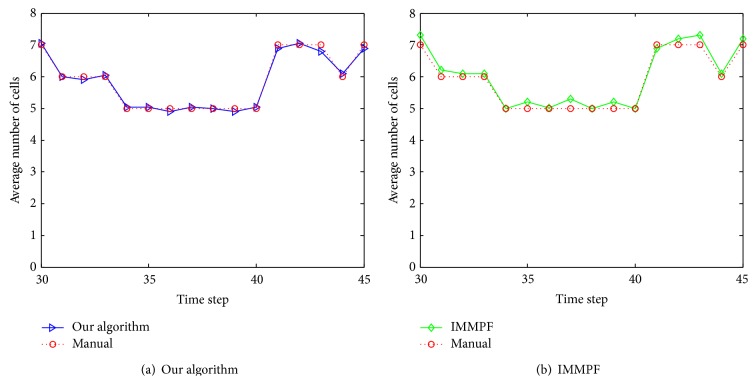
Comparison of cells number estimates by various modes.

Figure 14 shows the comparison of cells averaged number computed over 20 simulations by various methods. It can be seen that the cells averaged number estimated using our algorithm is close to the performance of the manual tracking method, whereas IMMPF may cause overestimated number of cells.

**Figure 15:**
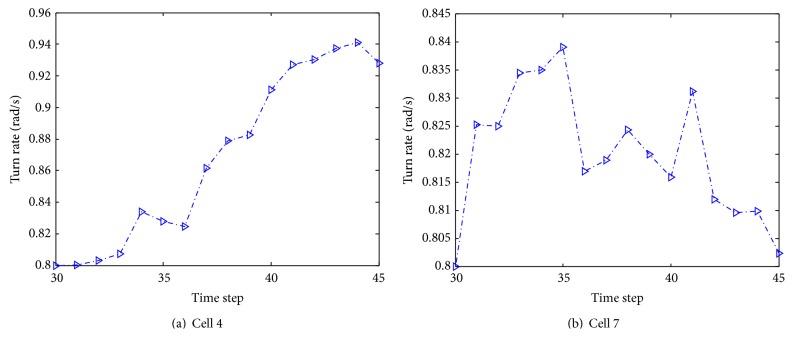
Results of turn rate estimate using our proposed algorithm.

**Figure 16:**
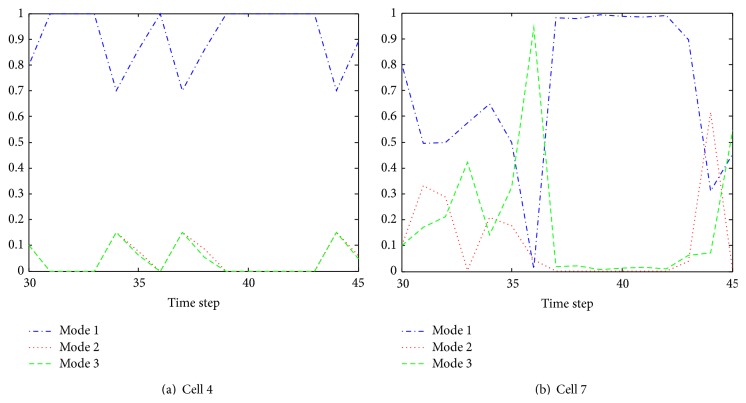
Mode probability of three modes.

**Figure 17:**
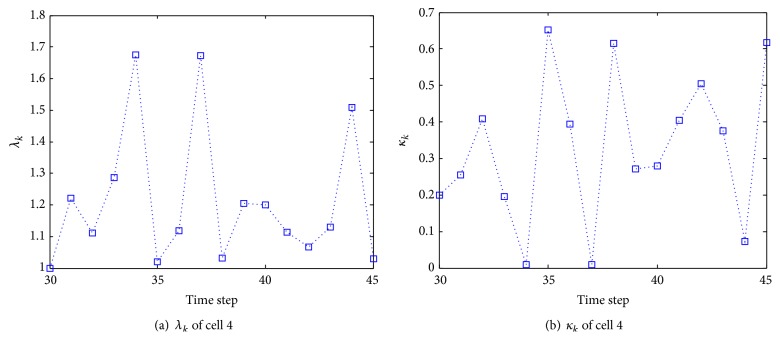
The evolving curve of variables *λ*_*k*_ and *κ*_*k*_ of cell 4.

Figure 15 shows the estimate of turn rate of selected cells 4 and 7 using our proposed algorithm. It can be seen that the cell turn rate changes over time. By comparing Figure 15(a) with Figure 11(b), it is obvious that when cell 4 colliding with other cells, turn rate undergoes significantly variations.  Figure 16 illustrates the mode probability of the three modes of cells 4 and 7. The blue dash dot line represents the mode probability of the mode ACV, the red dot line describes that of the mode ACT1, and the green dash line shows that of the mode ACT2. In theory, the mode probability change is related to the turn rate. The change of the mode probabilities in Figure 16 is the same as the change of the turn rate of the system described in Figure 15. It is obvious that the algorithm works well and the mode switching is right.  Figure 17 shows the evolving curve of *λ*_*k*_ and *κ*_*k*_ of cell 4 from frames 30 to 45. Observe that, in frames 34, 37, and 44, the variable *λ*_*k*_ of cell 4 is about 1.67, 1.67, and 1.51, respectively, which are larger than those in other frames because cell 4 occludes with cell 3. In frames 35, 38, and 45, the variable *κ*_*k*_ is larger than those in other frames because two cells split away one another.

**Scenario 2 .**

In this case, the event of cell division is investigated and the corresponding performance of our algorithm is evaluated with comparison to other methods as well. The Markov transition matrix between three modes is assumed constant and set empirically as $M=\left[\begin{array}{ccc} 0.8 & 0.1 & 0.1\\ 0.1 & 0.8 & 0.1\\ 0.1 & 0.1 & 0.8 \end{array}\right]$; *Q*_*k*,1_ = diag(50,50,0.1,0.1,0.01,0.01); *Q*_*k*,2_ = diag(80,80,0.1,0.1,0.01,0.01); *Q*_*k*,3_ = diag(40,40,0.1,0.1,0.01,0.01); *δ*_1_ = 5; *δ*_2_ = 5; $H_{k,•}={\left[\begin{array}{ccc} 1\;0\;0\;0\;0\;0 & 0\;1\;0\;0\;0\;0 & 0\;0\;0\;0\;0\;1 \end{array}\right]}^{T}$; *R* = diag(1,1, 1). The initial state of cell *i* is represented as $X_{0}^{}(i)={\left[x_{0}^{}(i),y_{0}^{}(i),{\dot{x}}_{0}^{}(i),{\dot{y}}_{0}^{}(i),\omega_{0}^{}(i),s_{0}^{}(i)\right]}^{T}$; here *x*_0_(*i*), *y*_0_(*i*), and *s*_0_(*i*) were determined by initial detected cells, and for other parameter initialization we set ${\dot{x}}_{0}^{}(i)=0.5\;\mu \text{m}/\text{s}$, ${\dot{y}}_{0}^{}(i)=0.5\;\mu \text{m}/\text{s}$, *ω*_0_(*r*) = 0.1 rad/s, *T* = 1, and *N* = 500. In the IMMPF algorithm, all parameters were set the same values as those in our proposed approach except *ω* = 0.1 rad/s, *λ*_*k*_ = 1.6, and *κ*_*k*_ = 0.5.

**Figure 18:**
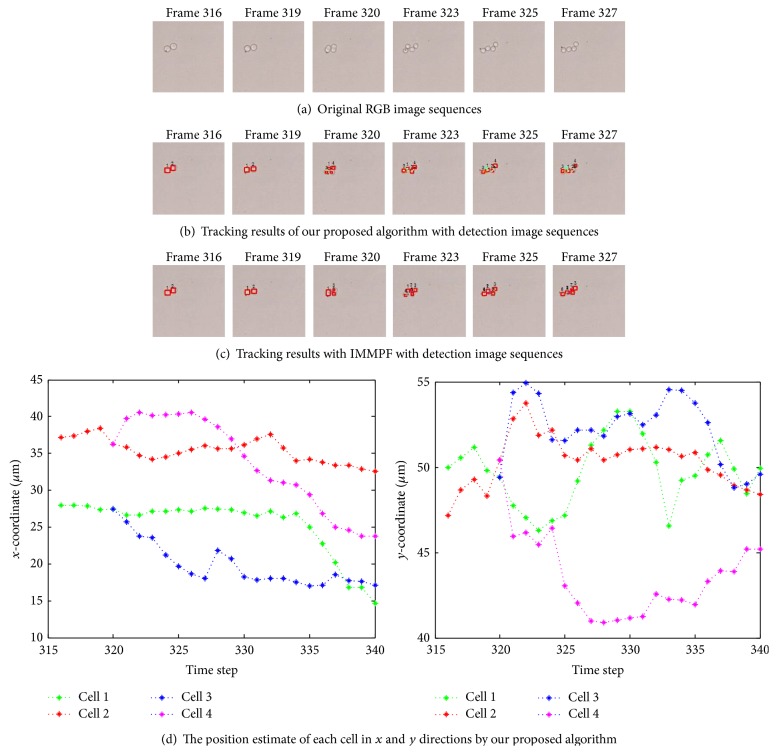
Tracking results on cell division using various methods.

As shown in Figure 18, there are two cells in initial frame, and cells 1 and 2 undergo sudden change and start dividing from frame 320; finally cell 1 divides into two cells (cells 1 and 3) and cell 2 divides into two cells (cells 2 and 4) completely in frame 323, respectively. Afterward, four cells are spatially adjacent and move slowly in the following frames. All cells are successfully tracked by our algorithm, as shown in Figure 18(b). The performance is degraded when the general IMMPF is used, as shown in Figure 18(c). Cells 1 and 2 are wrongly divided into five cells (cells 1 to 5) in frame 323 and two cells merge together as a cell in frame 325. A new cell 6 is wrongly initiated in frame 327.  Figure 18(d) gives the position estimates off our cells in each frame in *x* and *y* directions, respectively.

**Figure 19:**
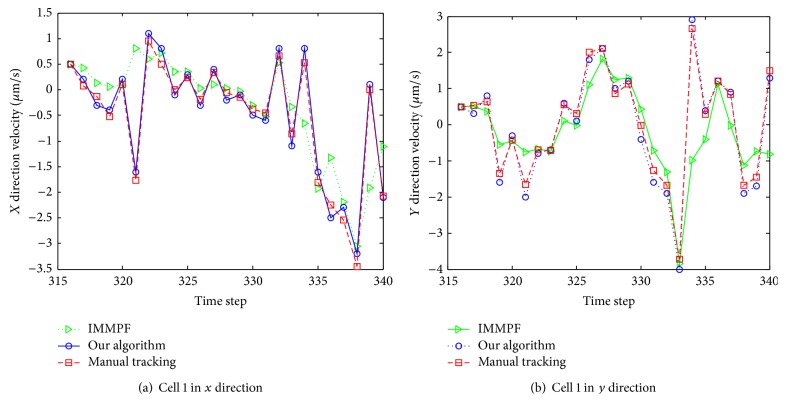
Instant velocity estimate per time step using various methods.

**Figure 20:**
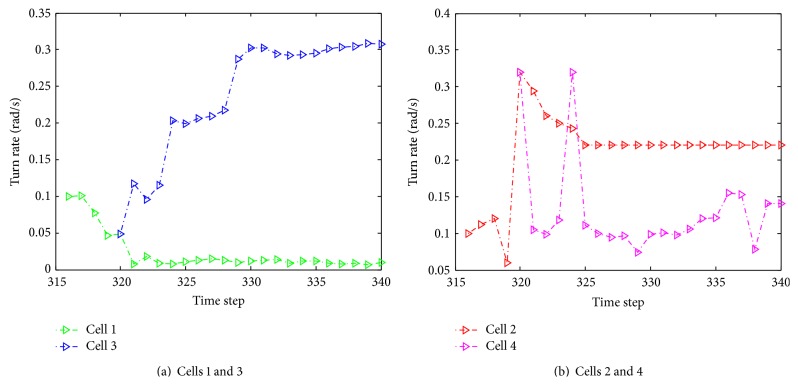
Results of turn rate estimate using our proposed algorithm.

**Figure 21:**
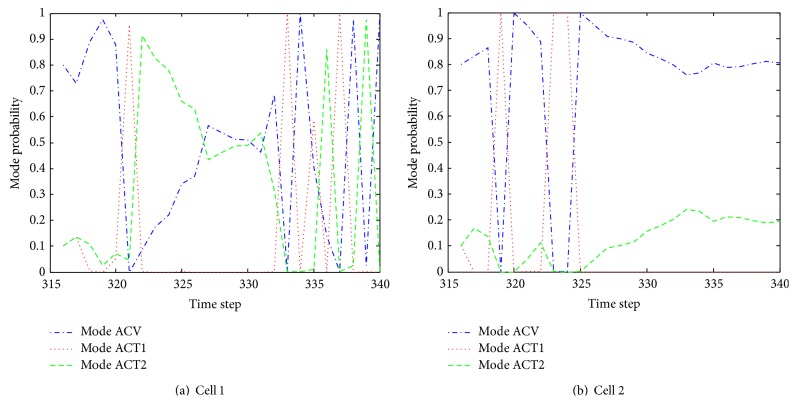
Model probability of three modes.

Instant velocity estimate for our proposed algorithm versus manual tracking is shown in Figure 19. The green dash dot line depicts the instant velocity of the IMMPF, the red dot line describes the instant velocity of the manual tracking, and the blue solid line shows instant velocity curves of our proposed algorithm. It is obvious that the instant velocity curve of our proposed algorithm fits the ground-truth data better than those of the IMMPF. Combining the results illustrated in Figures 18(b) and 18(c), our proposed algorithm could track* accurately* each cell in case of division due to the fact that instant velocity estimate errors using the IMMPF are larger than those using our proposed algorithm. Results of turn rate estimate using our proposed algorithm is shown in Figure 20. It is obvious that the cell turn rate changes over time.  Figure 21 shows mode probability of three modes, which indicates that the changes in mode probability coincide with the changes of turn rate estimate.

To get insight into tracking performance, we adopt one measure criterion, namely, percentage of tracked position (PAP) [33]. The PAP is defined as the ratio of the number of correctly tracked positions and the total number of ground-truth positions. All correctly track reports in each frame are recorded over 50 Monte-Carlo simulations, and the averaged values are listed in Table 1. According to the statistic results in Table 1, the averaged PTP are* 89.71%* and* 91.49%*, respectively, using our algorithm. The comparison results demonstrate that our algorithm outperforms the IMMPF method.

Real-time tracking is required in our studied multicell tracking approach, so the total processing time must be in principle less than the interval between consecutive samplings. Computation time using our method is only 3.1129 s and 1.1930 s for Scenarios 1 and 2 respectively, which is far less than the sampling interval *T* = 60 s for our studied image sequences. In this sense, our proposed method is applicable for automated cell real-time tracking.

## 4. Conclusions

In this paper, through introducing three types of interaction events among cells, namely, independence, collision, and division, an augmented interaction multiple models particle filter tracking algorithm has been presented for spatially adjacent cells with varying size. To reduce the ambiguity of correspondence and establish trajectories of interested cells, both cell topological feature and cell motion feature are used to manage data association problem. Simulation experiments on real image were carried out and the performance comparison has been reported. Our proposed algorithm can successfully track multiple cells of colliding, dividing, or cells of entering and/or leaving field of view, and so forth. Furthermore, it can provide accurate dynamic estimate of each cell, such as position, velocity, and turn rate.

## Figures and Tables

**Table 1:** Performance comparison results using two methods.

Method	PTP (Scenario 1)	PTP (Scenario 2)
IMMPF	77.94%	85.11%
Our method	89.71%	91.49%
